# Advanced therapeutic modalities in hepatocellular carcinoma: Novel insights

**DOI:** 10.1111/jcmm.16875

**Published:** 2021-08-23

**Authors:** Bahare Shokoohian, Babak Negahdari, Hamidreza Aboulkheyr Es, Manuchehr Abedi‐Valugerdi, Kaveh Baghaei, Tarun Agarwal, Tapas Kumar Maiti, Moustapha Hassan, Mustapha Najimi, Massoud Vosough

**Affiliations:** ^1^ Department of Medical Biotechnology School of Advanced Technologies in Medicine Tehran University of Medical Sciences Tehran Iran; ^2^ Department of Regenerative Medicine Cell Science Research Center Royan Institute for Stem Cell Biology and Technology ACECR Tehran Iran; ^3^ School of Biomedical Engineering University of Technology Sydney Sydney NSW Australia; ^4^ Laboratory Medicine Karolinska Institutet Experimental Cancer Medicine, Clinical Research Center (KFC), Novum Karolinska University Hospital‐Huddinge and Biomolecular and Cellular Medicine (BCM Stockholm Sweden; ^5^ Basic and Molecular Epidemiology of Gastrointestinal Disorders Research Center Research Institute for Gastroenterology and Liver Diseases Shahid Beheshti University of Medical Sciences Tehran Iran; ^6^ Department of Biotechnology Indian Institute of Technology Kharagpur India; ^7^ Laboratory of Pediatric Hepatology and Cell Therapy Institute of Experimental and Clinical Research Université Catholique de Louvain Brussels Belgium

**Keywords:** gene therapy, hepatocellular carcinoma, immune checkpoint inhibitors, immunotherapy, molecular‐targeted therapy, radionuclide therapy

## Abstract

Hepatocellular carcinoma (HCC), the most common type of liver cancer, is usually a latent and asymptomatic malignancy caused by different aetiologies, which is a result of various aberrant molecular heterogeneity and often diagnosed at advanced stages. The incidence and prevalence have significantly increased because of sedentary lifestyle, diabetes, chronic infection with hepatotropic viruses and exposure to aflatoxins. Due to advanced intra‐ or extrahepatic metastasis, recurrence is very common even after radical resection. In this paper, we highlighted novel therapeutic modalities, such as molecular‐targeted therapies, targeted radionuclide therapies and epigenetic modification‐based therapies. These topics are trending headlines and their combination with cell‐based immunotherapies, and gene therapy has provided promising prospects for the future of HCC treatment. Moreover, a comprehensive overview of current and advanced therapeutic approaches is discussed and the advantages and limitations of each strategy are described. Finally, very recent and approved novel combined therapies and their promising results in HCC treatment have been introduced.

## 1 BACKGROUND

According to the GLOBOCAN 2020 estimates of cancer incidence and mortality, liver cancer with approximately 905,677 (4.7%) new cases and 830,180 (8.3%) deaths annually is the sixth most commonly diagnosed cancer and the third leading cause of neoplastic disease‐related deaths worldwide. Moreover, in 2020, the American Cancer Society estimated 42,810 new cases and 30,160 deaths for liver and intrahepatic bile duct cancer in the US.^1^

Histologically, primary liver cancer can be divided into several subtypes based on cellular origin, including hepatocellular carcinoma (HCC) (comprising 75–85% of cases), intrahepatic cholangiocarcinoma (ICC) (comprising 10–15% of cases) and other rare forms.^2^ The major known risk factors for the development of HCC are chronic infection with hepatotropic viruses (mainly hepatitis B [HBV] and C [HCV] viruses), chemical irritation (alcohol abuse and aflatoxins), metabolic aberrations (diabetes and non‐alcoholic fatty liver disease, hereditary haemochromatosis) and immune‐related causes (cirrhosis‐associated immune dysfunction [CAID] syndrome and autoimmune hepatitis). Chronic irritation over two to four decades may lead to liver carcinogenesis. In most cases, HCC starts from small dysplastic lesions caused by a few mutations and progresses to the advanced form of the disease, which displays a wide molecular heterogeneity.^3^

Therefore, considering the complexity of HCC due to its heterogeneity and the involvement of various signalling pathways, adopting a similar treatment regimen for all patients would not be effective and could even exacerbate symptoms, a fact that led to the introduction of the term ‘personalized medicine’. However, because of the difficulties in allocating a unique treatment plan for each individual suffering from the ‘same’ disease, researchers have tried to classify patients into subpopulations based on their susceptibility to a particular disease or response to a specific treatment. This patient stratification has helped to tailor the treatment modalities for each individual. Such an approach has led to the US National Research Council's preference for the term ‘precision medicine’ rather than ‘personalized medicine’ in 2016.^4^, ^5^ Accordingly, different therapeutic approaches based on both molecular and cellular therapies have been developed. Molecular‐targeted therapy, targeted radionuclide therapy and epigenetic modification‐based therapies are therapeutic strategies at the molecular level that, in combination with cell‐based treatments including immunotherapy and gene therapy, and have provided promising results. This review discusses recent advances in the development of novel therapeutic approaches in the treatment of HCC.

## 2 MOLECULAR‐TARGETED THERAPY IN HCC (MULTI‐KINASE INHIBITORS)

**FIGURE 1:**
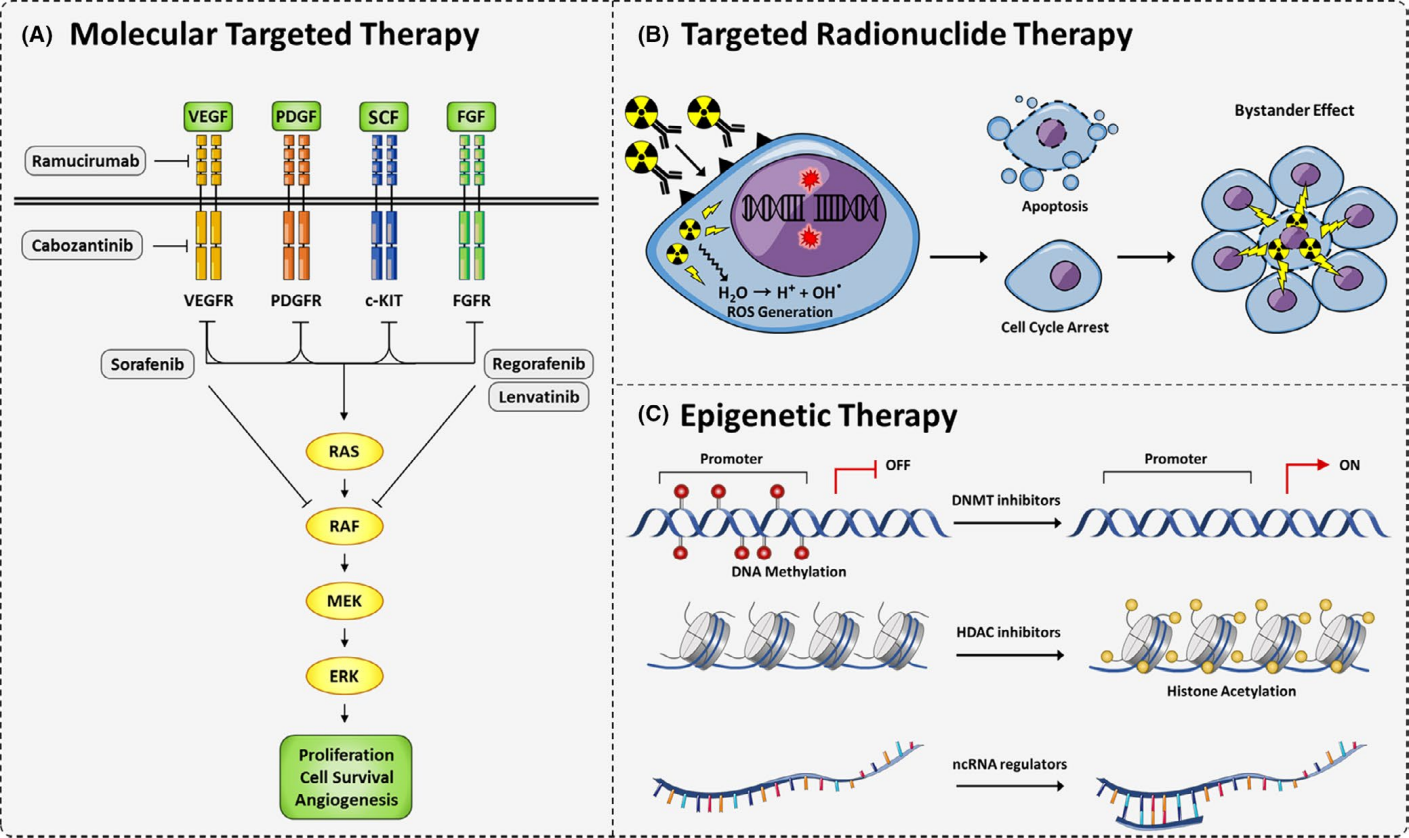
Schematic representation of molecular‐based therapies in HCC. (A) Molecular‐targeted therapies. Tyrosine kinase inhibitors and monoclonal antibodies inhibit their ligands and thereby prevent signalling pathways involved in cell proliferation and angiogenesis. (B) Targeted radionuclide therapy. Labelled radionuclides specifically target tumour cells and induce double‐stranded DNA breaks *via* water ionization. This effect can also eradicate neighbouring cells *via* a bystander effect. (C) Epigenetic alteration‐based therapies: DNMT and HDAC inhibitors and ncRNAs modulators return aberrant epigenetic alteration to the normal state. DNMT, DNA methyl transferase; HDAC, Histone deacetylase; ncRNA, non‐coding RNA

It is often observed that targeting a central biomolecule in a molecular network can affect the entire network and restore a large number of disrupted pathways and functions. This target can be a key transcription factor, a crucial receptor, or a central enzyme, such as a kinase (Figure 1A). However, targeting a single molecule (as what mono‐kinase inhibitors do) faces obstacles such as drug resistance development and weak potency. On the other hand, multi‐kinase inhibitors, which target several intracellular and cell surface kinases, can result in the pervasive inhibition of tumour proliferation, angiogenesis, metastasis and invasion. The approved medications in this context include sorafenib,^6^ and lenvatinib^7^ as the first‐line and regorafenib,^8^ cabozantinib^9^ and ramucirumab^10^ as the second line (Table S1).

## 3 TARGETED RADIONUCLIDE THERAPY IN HCC

The concept of targeted radionuclide therapy (TRT) relies on the use of injectable therapeutic radioisotopes designed to specifically target cancerous tissue at the cell or molecular level. The first application of radionuclides as therapeutic agents was demonstrated in the 1940s, when iodine‐131 (^131^I) was prescribed for treating thyroid diseases. Recent advances in radionuclide production and labelling as well as improvements in the identification of appropriate and specific molecular targets make the TRT an attractive approach for cancer treatment.^11^

Ionizing radiation interacts with biological substrates through direct and indirect mechanisms. Direct effects involve one‐electron oxidation reactions, while indirect effects are mediated by cytoplasmic water ionization, leading to the generation of reactive oxygen species (ROS). Radiation‐induced oxidative DNA damage (single‐strand breaks [SSB] and double‐strand DNA breaks [DSB], DNA base damage and disruption of DNA‐DNA or DNA‐protein interactions) may be caused by hydroxyl free radical (^●^OH) attack (indirect effect *via* water radiolysis) or by one‐electron oxidation (direct effect).^12^ The incidence of DNA damage is proportional to the absorbed dose and is quantified per grey (Gy) per cell. After exposure to radionuclides, DNA breaks can lead to apoptosis or cell cycle arrest in cancer cells. This destructive effect can be directed specifically towards the targeted cancer cells by conjugating a tumour‐specific ligand or antibody to the radionuclide, thus minimizing off‐target damage to the healthy tissues surrounding the tumour^13^, ^14^ (Figure 1B).

It is noteworthy that primary and metastatic liver lesions are highly vascularized and receive a preferential arterial supply *via* the hepatic artery, while normal liver cells are supplied at 80% by the portal vein. Accordingly, the hepatic artery is the appropriate route of administration for the delivery of targeted radionuclides.^15^, ^16^

### 3.1 Different approaches in targeted radionuclide therapy

Targeted radioembolization, using intra‐arterial Yttrium‐90 (^90^Y), Rhenium‐188 (^188^Re), Iodine‐125 (^125^I) and ^131^I, is a promising locoregional strategy for the treatment of HCC,^17^, ^18^, ^19^ and many intra‐arterial agents based on lipiodol‐labelled radionuclides have been developed so far.

Radioimmunotherapy is another approach that represents an advanced therapeutic modality for HCC using a combination of tumour‐specific antibodies with potent radiopharmaceuticals. This approach provides targeted radiation limited to the tumour cells with reduced side effects. HCC‐specific antigens such as PD‐1, PD‐L1, CTLA‐4, CD147 and endoglin (CD105) are potential targets for radionuclide antibody conjugates^20^, ^21^, ^22^, ^23^ (Table S2).

## 4 EPIGENETIC ALTERATION‐BASED THERAPIES IN HCC

Abnormal epigenetic alterations are important aetiologic factors in HCC initiation, progression and metastasis. Unlike the irreversible nature of genomic alterations, the reversibility of epigenetic changes opens a promising way forward for the development of new therapeutic modalities. The main epigenetic changes that have been studied in HCC are DNA methylation, histone modifications and the expression of non‐coding RNAs (Figure 1C).

HBV and HCV, as the leading causes of HCC, recruit DNA methyltransferases (DNMT1, DNMT3a and DNMT3b) to promote hyper‐methylation‐induced repression of tumour suppressor genes including *CDKN2A*, *SFRP1*, *SFRP5* and *CDH1*.^24^, ^25^

DNMT inhibitors can be divided into two categories based on their structure and function: nucleoside analogues and non‐nucleoside agents. Nucleosides, particularly cytosine analogues, such as 5‐azacytidine, 5‐aza‐2′‐deoxycytidine (decitabine)^26^ and zebularine,^27^ interact with, and block the activity of DNMTs, and decrease overall DNA methylation levels. Despite the promising preclinical data, these DNMT inhibitors have certain drawbacks; for example, cytidine deaminase, which is highly expressed in the liver, inactivates some cytosine analogues and reduces their half‐lives and efficacy in vivo.^28^ To overcome such limitations, next‐generation DNMT inhibitors such as guadecitabine (SGI‐110) that are resistant to cytidine deaminase degradation have been developed. Structurally, guadecitabine consists of decitabine and deoxy‐guanosine linked by a phosphodiester bond that can be cleaved gradually resulting in sustained demethylation.^29^

Moreover, different types of non‐nucleoside compounds, such as SGI‐1027,^30^ procaine,^31^ procainamide,^32^ hydralazine^33^ and EGCG,^34^ have been identified that act either through direct binding to the catalytic or cofactor site of DNMTs or by targeting their regulatory mRNA sequences.

The amino acid residues on the histone N‐terminal tails that protrude from the nucleosome cores may be subject to modifications including acetylation, methylation, phosphorylation and ubiquitination. The amino acid residues on the histone N‐terminal tails that protrude from the nucleosome cores may be subject to modifications including acetylation, methylation, phosphorylation and ubiquitination. The most studied histone modification is acetylation, a process regulated by a dynamic balance between the activities of two groups of enzymes: histone acetyltransferases (HATs) and histone deacetylases (HDACs). Loss of this balance has been reported in various diseases, including HCC.^35^, ^36^, ^37^

For example, HDAC1/2 and HDAC3, which have crucial roles in promoting tumour cell proliferation, invasion and metastasis, are often overexpressed in HCC, as demonstrated by several in vitro studies.^38^, ^39^ On the other hand, Patt1, an acetyltransferase with high expression in normal hepatocytes, is downregulated in HCC.^40^ Since the aberrant expression of HDACs is common in cancer patients, HDAC inhibitors are being widely considered in clinical trials.

Although the important role of non‐coding RNAs in various epigenetic regulatory processes has been extensively studied, only a few have entered the clinical phase in the treatment of HCC.

MTL‐CEBPA, the first small activating RNA drug, consists of liposomal nanoparticles that encapsulate CEBPA‐51, a 21‐mer small activating 2'‐O‐methylated double‐stranded RNA. It is designed to specifically target and upregulate transcription of the CEBPA gene which encodes a master regulator of liver homeostasis, and its expression is suppressed during liver carcinogenesis. It has been evaluated in a phase I trial on 24 patients with advanced HCC and results indicated an acceptable safety profile, 4% partial response rate and 50% stable disease.^41^ In another phase I clinical trial, MRX34, a liposomal formulation of miR‐34a as a tumour suppressor microRNA was evaluated. MiR‐34a expression is often lost or reduced in a broad range of cancers, including HCC. In normal tissue, miR‐34a suppresses the expression of MYC, PDGFR‐α, CDK4/6 and BCL‐2. Although the trial ended early due to unexpected severe immune‐mediated toxicities, dose‐dependent regulation of downstream genes could provide proof‐of‐concept for miRNA‐based cancer therapy^42^ (Table S3).

## 5 IMMUNOTHERAPY IN HCC

**FIGURE 2:**
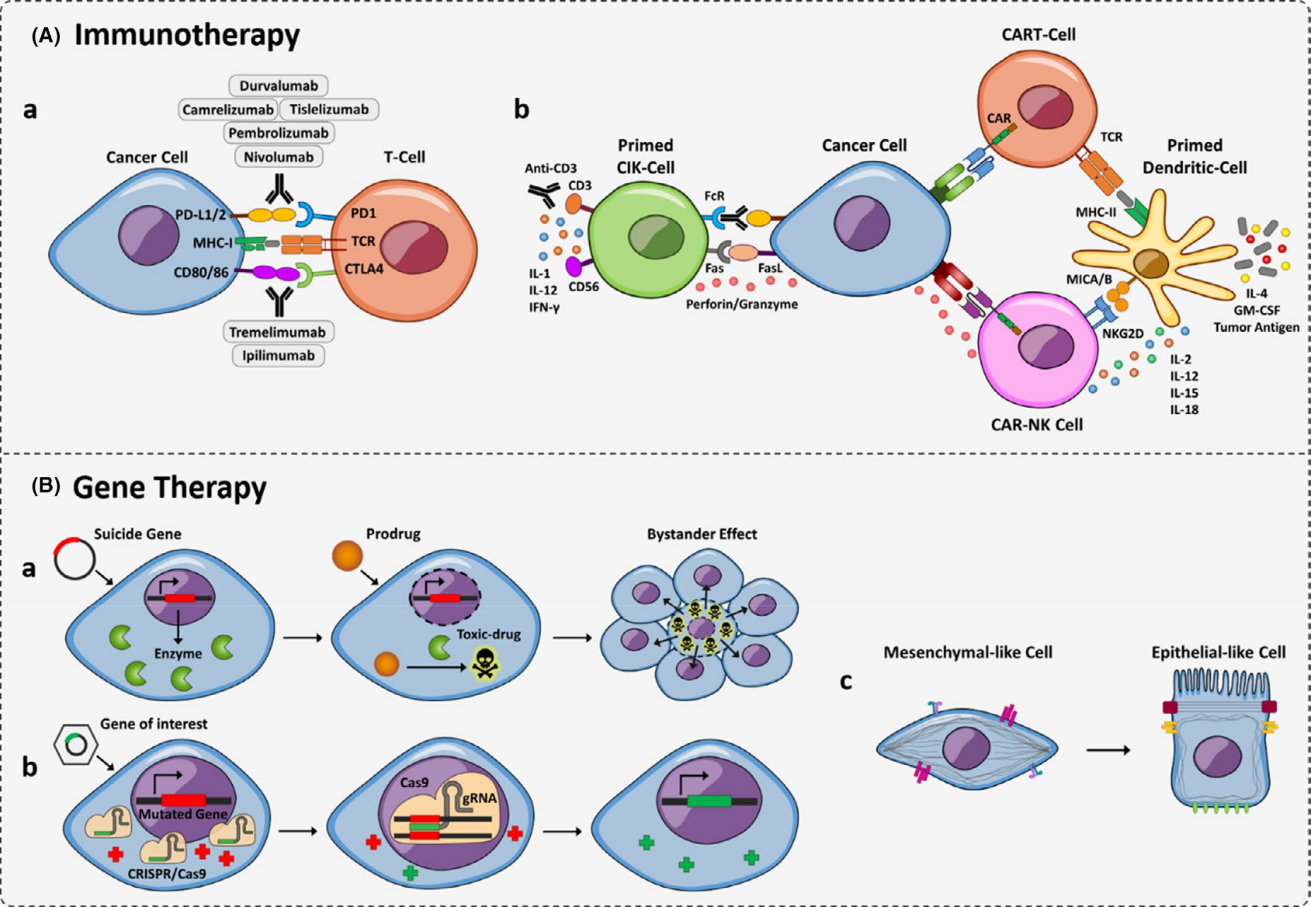
Schematic representation of cellular‐based therapies in HCC. (A) Immunotherapy in HCC treatment: a, Immune checkpoint inhibitors elevate the natural immunological response against cancer cells by blocking PD‐1/L1 and CTLA‐4; b, Immune cell‐based therapies: different cell types, including CIK, CAR‐T, CAR‐NK and dendritic cells are evaluated for HCC treatment. (B) HCC gene therapies: a, suicide gene therapy: introduction of a foreign enzyme converts a non‐toxic prodrug into a toxic anti‐metabolite, which can eradicate neighbouring cells *via* the bystander effect; b, gene replacement therapy: a mutated gene can be replaced with a normal gene; c. differentiation therapy: instead of ablating cancer cells, they can be returned to the differentiated and functional state

The chronic inflammatory state provides an opportunity for immune evasion by altering the expression profiles of inhibitory and stimulatory immune checkpoints (eg CTLA‐4, PD‐1, T‐cell immunoglobulin and mucin domain‐3 [TIM‐3], lymphocyte activation gene‐3 [LAG‐3] and glucocorticoid‐induced tumour necrosis factor receptor [GITR]), increasing the number of regulatory T cells (T‐regs), altering the function of dendritic cells (DCs) and releasing immuno‐modulating cytokines (such as IL‐10 and TGF‐β). Thus, in HCC and other cancers that are unresponsive to conventional therapies, immunotherapeutical approaches might be beneficial^43^ (Figure 2A).

### 5.1 Immune checkpoint inhibitors

Immune checkpoints are a sub‐type of membrane‐bound protein involved in triggering pivotal inhibitory and stimulatory pathways and are effective in the maintenance of self‐tolerance. In cancer, the aberrant activation of immune checkpoint pathways inhibits the anti‐tumour immune response. Several trials based on immune checkpoint therapy have attempted to block or stimulate the function of these pathways in HCC and thereby enhance the body's immunological reaction against tumours. The most studied immune checkpoint effectors in HCC include CTLA‐4, PD‐1 and PD‐L1, TIM‐3 and LAG‐3^44^, ^45^ (Figure 2A).

#### 5.1.1 Anti‐CTLA‐4 (tremelimumab, ipilimumab)

CTLA‐4, a CD28 homolog, is present on cytotoxic T cells and recognizes the same ligands (B7‐1 and B7‐2) as CD28 but has a higher binding affinity towards them. It prevents co‐stimulation, which would normally be provided *via* the CD28‐B7 interaction, by outcompeting CD28. In the early phase of tumorigenesis, CTLA‐4 can attenuate the immune response by producing inhibitory signals.^46^

Tremelimumab (CP‐675,206), a fully human IgG2 antibody against CTLA‐4, was the first molecule to be clinically tested for safety and efficacy in HCC.^47^ Twenty patients with advanced HCC developed from HCV‐induced liver cirrhosis, who were not eligible for surgery or locoregional therapy, were treated with a suboptimal dose of the tremelimumab in a phase II clinical trial (NCT01008358). In general, the treatment was well‐tolerated and not only showed anti‐tumour activity (partial response rate of 17.6% and disease control rate of 76.4%), but also a significant drop in viral load, suggesting an anti‐viral effect of the immune checkpoint blockade.^23^

Ipilimumab is another important anti‐CTLA‐4 IgG1 antibody that was approved by the FDA as an anti‐melanoma agent in March 2011, by the EMA in July 2011 and by Japan PMDA in 2015 (67). It is currently being evaluated in combination with nivolumab for the treatment of HCC as part of the CheckMate‐040 trial (NCT01658878).

#### 5.1.2 Anti‐PD‐1/L1 (nivolumab, pembrolizumab, tislelizumab, camrelizumab and durvalumab)

PD‐1 is an immune co‐inhibitory receptor that is expressed mainly on T cells at the late activation stage and plays a vital role in maintaining immune tolerance. PD‐1 interacts with its ligands, PD‐L1 and PD‐L2, which are expressed on Kupffer cells, sinusoidal endothelial cells, hepatocytes and stellate cells.^43^

An increase in the population of PD‐1^+^/CD8^+^ T cells is associated with the progression of HBV‐related hepatic cirrhosis to HCC, high post‐operative recurrence and poor prognosis. Upregulation of PD‐L1 is induced by a variety of cytokines, particularly IFN‐γ, during chronic viral infection and other inflammatory disorders, which leads to the impairment of anti‐tumour immunity and induces apoptosis in CD8^+^ T cells.^48^

Nivolumab is the first recombinant human IgG4 mAB against human PD‐1. It prevents suppression of the nascent anti‐tumour immune response by blocking PD‐1 on the surface of CD8^+^ T cells.^49^

**FIGURE 3:**
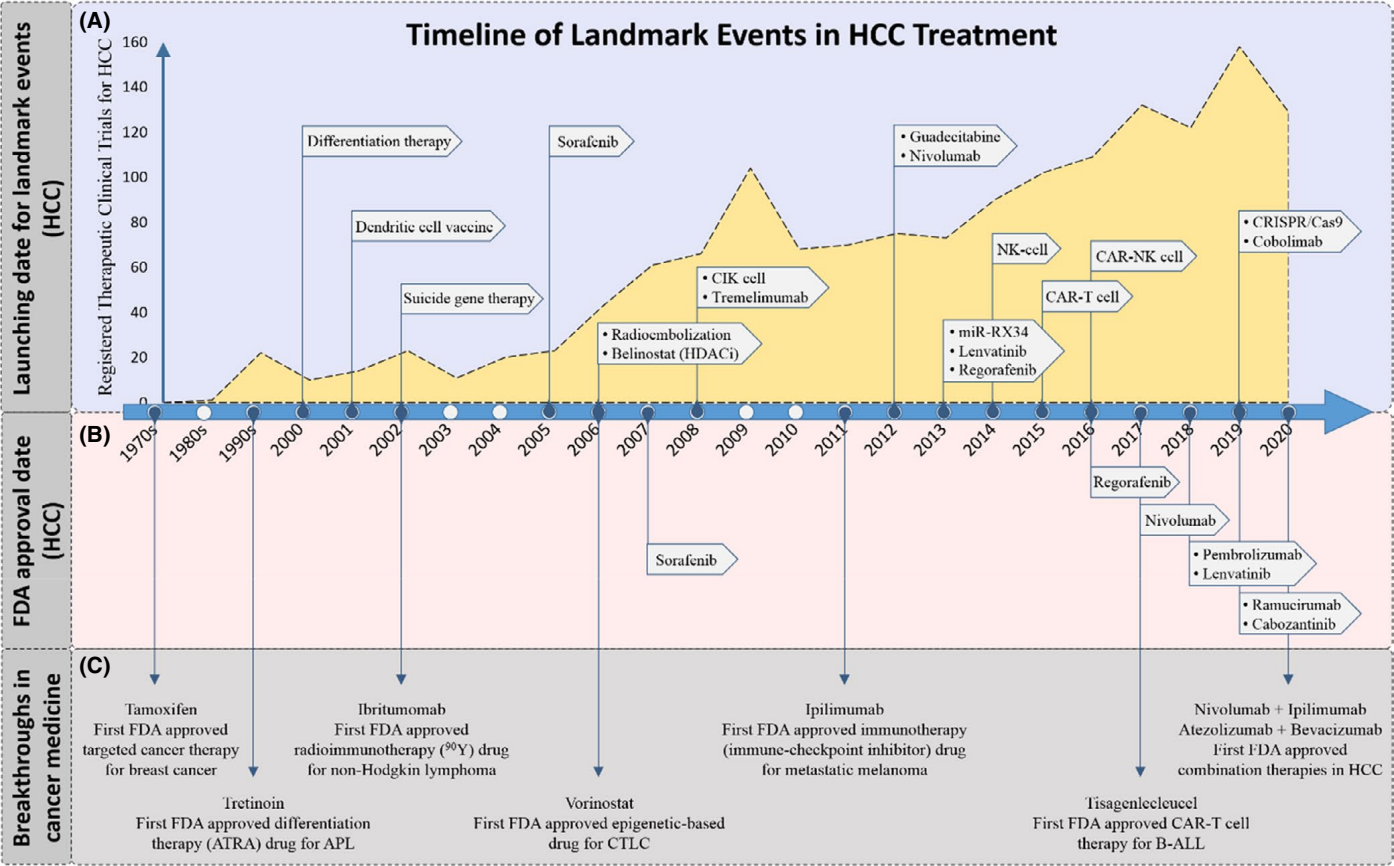
Timeline of landmark events in cancer and HCC treatment. (A) In the upper box, flags indicate the start time of the key clinical trials as well as the first submitted clinical trial for each HCC treatment modality. The upward slope represents the increasing number of registered trials for HCC treatment using novel modalities in each year. (B) The middle box shows the FDA approval dates for novel medications prescribed for HCC treatment. (C) The bottom box indicates the first, FDA‐approved landmark medications in different cancer therapeutic approaches

Nivolumab was evaluated in CheckMate‐040, a clinical trial involving 154 patients with advanced HCC who were intolerant or refractory to sorafenib. After sorafenib, nivolumab gained FDA approval in 2017 as a second‐line agent after a successful report of a 20% response rate and 64% disease control^50^ (Figure 3). These results encouraged researchers to initiate two ongoing phase III trials: (i) CheckMate‐459 (NCT02576509) to compare nivolumab against sorafenib as a first‐line agent and (ii) CheckMate‐9DX (NCT03383458) to evaluate nivolumab against placebo as an adjuvant therapy in patients at higher risk of recurrence after tumour resection or ablation.

Pembrolizumab is a fully human IgG4 mAB targeting PD‐1. This antibody demonstrated its effectiveness and tolerability in two distinct phase II trials. One of these, KEYNOTE‐224 (NCT02702414), an open‐label, phase II trial enrolled 104 patients with advanced HCC who had been treated with sorafenib and reported a response rate of 17% and 54% OS after 12 months. The results indicated that the effects were comparable with those of nivolumab.^51^ Because of the promising results, the FDA also approved pembrolizumab in November 2018, as a second‐line therapy for patients who were refractory to sorafenib (Figure 3). In February 2019, Merck announced preliminary data from a confirmatory phase III trial (KEYNOTE‐240/ NCT02702401) of 413 patients with a history of systemic therapy for HCC; however, the KEYNOTE‐240 trial did not meet the co‐primary endpoints for extending PFS and OS.^52^

Tislelizumab (BGB‐A317) is another high‐affinity anti‐PD‐1 IgG4 antibody; however, preclinical data suggest that this antibody does not interact with FcγR‐1 on macrophages. Therefore, tislelizumab not only shields CD8^+^ T cells from PD‐L1 interference but also protects T cells against the activation of ‘antibody‐dependent macrophage‐mediated killing of effector T cells’.^53^ After safety confirmation in a phase I trial, a global phase III trial (NCT03412773) was started in December 2017 to evaluate the efficacy, safety and non‐inferiority of tislelizumab compared to sorafenib, as a first‐line systemic treatment, in patients with unresectable HCC.

Camrelizumab (SHR‐1210) is an IgG4 mAB against PD‐1. A different binding epitope for camrelizumab, compared to nivolumab and pembrolizumab, has led to greater occupation of receptors on circulating T lymphocytes. A phase II/III trial (NCT02989922) is underway in China on patients who failed to respond, or were intolerant, to systemic treatment. The results suggest that camrelizumab has anti‐tumour activity, preliminary survival benefit, a manageable safety profile and might be a potential second‐line treatment for advanced HCC.^54^

Durvalumab, unlike the others, is an anti‐PD‐L1 IgG1 mAB that selectively blocks PD‐L1 binding to PD‐1 and CD80 (B7‐1). Durvalumab, like tislelizumab, has a triple mutation in the Fc domain and does not induce antibody‐dependent cell‐mediated cytotoxicity (ADCC).^55^ A phase I/II trial (NCT01693562) of durvalumab monotherapy for solid tumours, which enrolled 40 patients with advanced HCC, has been completed and reported 10% response rate and 13.2 months median OS in the treated population. Notably, the tumour response to durvalumab occurred early and was durable.^56^

#### 5.1.3 Other inhibitory and co‐stimulatory immune checkpoints

Despite the promising clinical success of immune checkpoint therapy, tumour‐intrinsic resistance remains an unsolved challenge and has led researchers to target alternative molecules in the tumour microenvironment (TME). Immune checkpoint molecules, such as TIM‐3, LAG‐3 and GITR, have also been identified in HCC and are associated with a poor prognosis.

TIM‐3, a transmembrane protein, is mainly expressed on IFN‐γ‐secreting Th1 cells, natural killer (NK) cells and CTLs and plays a crucial role in inhibiting both the adaptive and innate immune responses. TIM‐3 interacts with its soluble ligand, galectin‐9, which is highly expressed by antigen‐presenting cells (APCs) in HCC, to inhibit anti‐tumour immunity by mediating inducing T‐cell exhaustion and senescence. The expression of TIM‐3 is often increased in infiltrating T cells in chronic HBV‐infected liver, and the TIM‐3/galectin‐9 pathway consistently predicts a poor prognosis in patients with HBV‐associated HCC.^57^ A phase II trial (NCT03680508) investigating the combined effects of cobolimab (TSR‐022, an anti‐TIM‐3 agent) and dostarlimab (TSR‐044, an anti‐PD‐1 antibody) in patients with locally advanced or metastatic liver cancer is already in progress. Previous studies have shown that an anti‐TIM‐3 antibody, by binding to TIM‐3 expressed on tumour‐infiltrating lymphocytes (TILs) such as CD8^+^ T cells, reduces tumour growth *via* the induction of cytotoxic T‐cell‐mediated tumour cell lysis.^58^

LAG‐3, a member of the immunoglobulin superfamily, which often binds to major histocompatibility complex‐II (MHC‐II) molecules with high affinity, prohibits binding of the same MHC molecule to the T‐cell receptor (TCR) and CD4.^59^ In HCC patients, LAG‐3 plays an inhibitory role in the functionality of HBV‐specific CD8^+^ T cells, which are derived from TILs *via* selective upregulation.^60^ Clinically, a dual blockade of LAG‐3 (relatlimab) and PD‐1 (nivolumab) is currently being tested in a phase I trial (NCT01968109), and their clinical benefits await further clarification.

The activation of signalling pathways mediated by co‐stimulatory immune checkpoint molecules, such as GITR, is another remarkable approach. GITR is a member of the tumour necrosis factor (TNF) receptor superfamily whose expression rapidly increases on both regulatory and effector T cells following activation through two distinct signalling pathways, suggesting a cell type‐specific regulation of expression. In HCC, GITR binding enhances the proliferation and tumour antigen‐specific stimulation of TILs isolated from tumours.^61^ Recently, a phase I/II trial (NCT04021043) has started, focussing on an anti‐GITR agonist (BMS‐986156) along with ipilimumab and nivolumab for treating metastatic lung and liver cancers. BMS‐986156 can induce the activation and proliferation of effector T cells and at the same time suppresses the function of activated regulatory T cells.^61^

### 5.2 Immune cell‐based therapies

In addition to the immune checkpoint blockade, different types of cell‐based therapies involving cytokine‐induced killer (CIK) cells, chimeric antigen receptor T cells (CAR‐T) or NK cells (CAR‐NK), and DCs, are also being considered and evaluated for the treatment of HCC^62^ (Figure 2A).

#### 5.2.1 Cytokine‐induced killer (CIK) cells

CIK cells are a heterogeneous subset of immune effector cells that can be expanded *ex vivo* in the presence of IL‐1, IL‐2, IFN‐γ and an anti‐CD3 antibody. These cells include activated CD3^ˉ^/CD56^+^ NK cells, CD3^+^/CD56^+^ natural killer T (NKT) cells and CD3^+^/CD56^ˉ^ T cells, which display a non‐MHC restricted cytotoxicity against a broad range of cancer cells.^44^ A randomized phase II trial demonstrated the advantages of CIK cells in non‐surgical HCC patients. The trial revealed that receiving CIK cells, along with standard treatment, could significantly increase the median OS and PFS at the end of one, two, and three years.^63^ A phase III clinical trial (NCT00699816) on the efficacy and tolerability of CIK cell treatment in 230 patients with HCC confirmed that adjuvant immunotherapy with CIK cells could improve the PFS from 30 to 44 months and was associated with non‐significant serious adverse events compared to the control group.^64^ These early promising results have led to a flood of studies with 13 ongoing clinical trials on CIK cells in HCC.

#### 5.2.2 Chimeric antigen receptor T cells

Another adoptive cell‐based immunotherapy that is mainly based on achievements in CD19^+^ haematological malignancies has gained much attention for the treatment of HCC. This technology uses T cells with CAR cells or genetically modified NK cells. Chimeric T‐cell receptors are assembled from an extracellular antigen recognition domain, which is derived from an anti‐tumour associate antigen (anti‐TAA) mAB, connected to the transmembrane and intracellular signalling and activation domains. CAR‐T cells can target specific antigens accurately in an MHC‐independent manner.^65^

In HCC, glypican‐3 (GPC3), an oncofetal proteoglycan, is a TAA used as the CAR‐T‐cell therapy target in three completed clinical trials (NCT02395250, NCT02723942 and NCT03146234) and at least 11 ongoing studies.^66^ Moreover, other tumour antigens have also been identified in malignant liver cells, including AFP,^67^ CD133,^68^ c‐Met (NCT03672305), EpCAM (NCT03013712), DR5 (NCT03941626) and MUC‐1 (NCT02587689) and are being studied in different phases of various clinical trials.

Despite promising results, CAR‐T‐cell‐based therapy in solid tumours has faced many hindrances. Finding the TAAs that have the highest expression on tumour cells is the first challenge, to minimize off‐target reactions. Additionally, the spatiotemporal composition of cells within the TME and tumour immune microenvironment (TIME) of solid tumours favours the tumour cells. The impenetrability of the tumour mass and insufficient penetration of T cell into the tumour core, along with the immunosuppressive microenvironment, are other challenges that justify a large number of further studies in this field.^69^

#### 5.2.3 Natural killer cells

NK cells have a particular phenotype and function that make them a desirable treatment modality for a variety of cancers, especially HCC. Intrahepatic NK cells, which make up 30–50% of liver leukocytes, have a higher cytotoxicity against cancer cells compared to circulating NK cells. In addition, during liver carcinogenesis, the frequency and cytotoxic activity of NK cells diminishes, which is associated with the relapse and reduced survival rate of patients with resectable HCC.^70^

In addition to other approaches for employing NK cells, CAR‐modified NKs have more advantages than CAR‐T cells, in cancer treatment. CAR‐NK cells have a shorter lifespan than CAR‐T cells, which reduces the risk of autoimmunity or neoplastic transformation. Moreover, cytokines secreted by NK cells, such as IFN‐γ and GM‐CSF, are safer and avoid the cytokine storm that may result from CAR‐T‐cell therapy.^71^

A pioneering study revealed that human IL‐15 gene‐modified NKL cells (NKL‐IL‐15) could increase the susceptibility of HCCs to NKL‐mediated cytolysis *via* the expression of cytolysis‐related molecules (NKp80, TRAIL, granzyme B, IFN‐γ and TNF‐α) and the induction of NKG2D ligand overexpression on tumour cells.^72^ GPC3‐specific CAR‐NK‐92 cells were also indicated to have an effective cytokine production and anti‐tumour activity on HCC xenografts, expressing both low and high levels of GPC3, while they were not reactive to GPC3‐negative cells. Additionally, hypoxic conditions (1% O_2_), which are typically present in solid tumours and act as an immunosuppressive agent, did not significantly affect the anti‐tumour activity of CAR‐NK‐92 cells.^73^ A recent study using HepG2 cells, as an in vitro HCC model, demonstrated that c‐Met‐specific CAR‐NK cells have specific and more potent cytotoxicity against c‐Met‐positive HepG2 cells than the lung cancer cell line, H1299, with lower c‐Met expression.^74^ A phase I/II clinical trial (NCT02839954) is underway to evaluate the safety and effectiveness of anti‐MUC‐1 CAR‐pNK cells in MUC‐1 positive refractory solid tumours, including liver cancer.

#### 5.2.4 Dendritic cells

DCs, the most potent antigen‐presenting cells, play a major role in priming the anti‐tumour immune response *via* the recruitment of T and NK cells. However, particular tumour conditions, such as limited access to TAAs, prevent DCs from functioning properly.^75^ Bioengineered DCs with toll‐like receptor (TLR) agonists, TAAs, TAA‐derived peptides and tumour lysates have recently been developed as novel vaccines and are broadly used in treating breast cancer,^76^ lung cancer,^77^ prostate cancer^78^ and HCC.^79^, ^80^, ^81^

A phase I/II clinical trial assessed intradermal immunization with DC‐based vaccines. Four AFP peptides were pulsed *ex vivo* onto autologous DCs, which resulted in an increase in the IFN‐γ‐producing AFP‐specific T‐cell response.^79^ A similar study tested the safety and efficacy of a multiple TAA‐pulsed DC vaccine with AFP, GPC3 and MAGE‐1 recombinant fusion proteins.^80^ In a phase I trial, ilixadencel, activated allogeneic DCs using a combination of proinflammatory factors, was evaluated as a single agent and combined with sorafenib in patients with advanced HCC.^81^ Results revealed an increase in the frequency of CD8^+^ T cells in 73% of enrolled patients.

Another strategy is to administer artificially induced DCs (by GM‐CSF and IL‐4) to effectively boost DC‐mediated host immune responses.^82^ DC‐derived membrane vesicles or exosomes (DEXs) are a new class of cell‐free vaccines for cancer immunotherapy.^83^ Exosomes, derived from AFP‐expressing DCs (DEX_AFP_) in mouse models, revealed an efficient triggering of antigen‐specific immune responses, substantial tumour growth retardation and increased survival rates. In addition, the TME underwent extensive alterations, including an increase in the number of IL‐2 and IFN‐γ‐expressing CD8^+^ T lymphocytes and a decrease in IL‐10, TGF‐β and regulatory T cells.^84^ Despite the current challenges and novelty of the field, the inadequate number of studies in DC‐based HCC immunotherapy indicates that this area still needs further investigation.

## 6 GENE THERAPY IN HCC

Gene therapy is the delivery of therapeutic genetic materials into affected cells in order to correct a genetic abnormality or restore a missing function. In this context, scientists have faced two main challenges: an optimal delivery method and the manipulation strategy.^85^ Among the delivery methods, utilizing replication‐deficient modified viruses is the most widely used technique in the clinical trials (Table S4).

### 6.1 Gene therapy strategies

#### 6.1.1 Suicide gene‐based therapy

Suicide genes (prodrug transforming genes) are transgenes that encode an enzyme with the ability to convert a non‐toxic prodrug substrate into a toxic anti‐metabolite able to halt the synthesis of nucleic acids and resulting in the death of host cells. Rationally, even under optimal conditions, only a small percentage of tumour cells would receive the transgene. However, the toxic metabolite, produced in this small number of modified cells can enter neighbouring non‐transduced cells through cell junctions and result in the complete eradication of cancer cells, a phenomenon known as the ‘bystander effect’. However, low transfection/transduction efficiencies and slow conversion rates of prodrug to drug are avenues for further exploration.^86^

So far, various suicide gene therapy or gene‐directed enzyme/prodrug therapy (GDEPT) systems have been developed. Among them, the most commonly used suicide gene/prodrug pairs in HCC treatment include herpes simplex virus thymidine kinase/ganciclovir (HSVtk/GCV), cytosine deaminase/5‐fluorocytosine (CD/5‐FC) and purine nucleoside phosphorylase/fludarabine phosphate (PNP/FP)^87^ (Table S5).

HSVtk/GCV is the first and most commonly used suicide gene therapy system. HSVtk phosphorylates GCV (a guanosine analogue) to GCV monophosphate, which is followed by two more phosphorylation reactions by endogenous kinases, thereby converting it to GCV‐triphosphate. Its incorporation during DNA replication leads to single‐strand DNA breaks, inhibition of DNA polymerase, DNA chain termination and finally apoptosis induction. Despite the positive results of this system in several studies, there are some limitations, such as relatively slow kinetics, immunogenicity due to viral origi, and non‐specific toxicity. Furthermore, HSVtk/GCV mainly exerts its bystander effect *via* gap junctions, while in tumours with cystic and necrotic regions cell‐cell interactions may be reduced or lost, which drastically decreases the efficiency of this treatment.^88^

CD/5‐FC is another suicide gene therapy with a similar rudimentary operating principle to other systems. The bacterial or yeast cytosine deaminase converts 5‐fluorocytosine to 5‐fluorouracil (5‐FU), a highly toxic and diffusible metabolite, which is also used as a chemotherapeutic agent and radiosensitizer. It can exhibit a strong bystander effect, due to its small size and neutral charge. Thus, the use of the CD/5‐FC system in combination with ionizing radiation, in in vitro and in vivo investigations, has shown more efficient cell ablation responses in comparison to the individual effect of each.^89^

The *E*.*coli* PNP can convert some adenosine analogues, such as 6‐methylpurine 2‐deoxyriboside (MeP‐dR) and fludarabine, to adenine compounds. Conversion of fludarabine phosphate (FP) to its active metabolite, 2‐fluoroadenine by PNP and then to 2‐fluoroadenine triphosphate by intracellular kinases, generates a replication and transcription terminator, which can eradicate both dividing and non‐dividing cells. More importantly, 2‐fluoroadenine triphosphate is freely diffusible across cell membranes and also takes advantage of nucleoside transporters, resulting in a potent bystander effect^90^ (Figure 2B).

#### 6.1.2 Gene replacement therapy

The modification of mutated priming genes in cancer cells is another strategy that faces limitations such as the heterogeneity of tumour cells and the need for a high‐throughput method to avoid skipping malignant cells. Many molecular gene editing tools, based on programmable nucleases such as meganucleases (MN), zinc finger nucleases (ZFN) and transcription activator‐like effector nucleases (TALEN), have been developed; however, the most notable of them currently is CRISPR/Cas9.

The CRISPR/Cas9 system consists of three components: an endonuclease (Cas9), a sequence‐specific targeting element (crRNA) and tracrRNA that pairs with crRNA and guides Cas9. Recently, crRNA and tracrRNA were combined in a single RNA called single guide RNA (sgRNA). Bioengineered CRISPR/Cas9 systems can create a double‐stranded break in the target DNA sequence, which is mainly repaired by the non‐homologous end joining (NHEJ) pathway. This error‐prone system, which can result in insertions or deletions, thus potentially disrupting the open reading frame, is used for creating loss‐of‐function (knockout) mutations in the gene of interest. Double‐strand breaks can also be repaired by homology‐directed repair (HDR), which needs a donor or template DNA and enables the precise insertion of new sequences that could be used to correct mutated genes.^91^

For example, in order to block angiogenesis after trans‐arterial embolization (TAE), hypoxia‐inducible factor‐1α (HIF‐1α) knockout was generated using a lentivirus‐mediated CRISPR/Cas9 system. The HIF‐1α knockout in SMMC 7721 cells significantly inhibited cell invasiveness and migration and prolonged the survival of HCC‐bearing mice.^92^ In another report, it was shown that a CRISPR/Cas9‐mediated NSD1 knockout suppressed HCC cell proliferation, migration and invasion *via* the Wnt/β‐catenin signalling pathway^93^ (Figure 2B).

#### 6.1.3 Differentiation therapy

Evaluation of the transcriptome of cancer cells has revealed the re‐expression of some embryonic genes that were downregulated or silenced during the differentiation/maturation process. Such alterations in gene expression patterns are gradually reflected in the morphological and proliferative features of cells, a phenomenon known as the epithelial‐mesenchymal transition (EMT). Epithelial cells, by losing their polarized polygonal shapes along with a reduction in cell‐cell and cell‐extracellular matrix (ECM) interactions, transform into a mesenchymal‐like phenotype, characterized by their spindle shape and the ability to migrate and invade.^94^, ^95^

EMT and its reverse process, mesenchymal‐epithelial transition (MET), are the cornerstone of a hypothesis which suggests that neoplasia is a cell differentiation disorder.^96^ Accordingly, the differentiation therapy hypothesis was proposed, suggesting that instead of ablating malignant cells, the previous well‐differentiated status can be restored and normal function reactivated.^97^ Therefore, all agents that can induce cell differentiation are theoretical treatment options, such as all‐trans‐retinoic acid (ATRA), which is often prescribed to treat acute promyelocytic leukaemia (APL) with excellent results.^98^

There are several approaches in HCC differentiation therapy, including targeting the regulation of deviated transcription factors, miRNA profile pattern and signalling pathways. Many studies have tried to upregulate HNF4α and HNF1α as the crucial transcription factors in hepatocyte maturation and differentiation. It is known that re‐expression of HNF4α decreases the percentage of cells expressing CD133, a cancer stem cell marker. Meanwhile, HNF4α induced apoptosis in Hep3B and senescence in HepG2 cells and abolished tumorigenesis in a mouse model.^99^ Similar results were obtained with HNF1α overexpression, which restored the expression of miR‐192 and miR‐194 levels, with a subsequent increase in p21 levels and the induction of cell cycle arrest at the G2/M stage in vivo and in vitro.^100^ In addition, a notable study indicated that treatment with siRNA‐lipid nanoparticles (siRNA‐LNPs) targeting YAP, a transcription co‐activator involved in cell proliferation and differentiation, restored hepatocyte differentiation and led to tumour regression in an HCC mouse model.^101^

On the other hand, regulation of miR‐122, the most abundant liver‐specific miRNA, adjusted the liver‐enriched transcription factor network and re‐established the expression of hepatocytic genes.^102^, ^103^ The microRNA, miR‐148a, was identified as a hepatocyte differentiation inducer through inhibition of the NOTCH signalling pathway.^104^ Moreover, it has been shown that certain macromolecules can inhibit EMT or even induce MET by targeting cancer stem cell‐related pathways.^105^, ^106^

Altogether, it is clear that differentiation therapy as a non‐invasive treatment or a complementary approach alongside conventional therapies needs more consideration in clinical studies (Figure 2B).

## 7 HOW FAR WE HAVE COME, HOW FAR WE STILL HAVE TO GO?

HCC is typically a latent and asymptomatic malignancy with different aetiologies, which result in a wide molecular heterogeneity and is often diagnosed at advanced stages. Due to advanced intra‐ or extrahepatic metastasis, conventional treatments are inefficient and the implementation of multidisciplinary treatments is strongly recommended.

In this review, the advantages and limitations of five novel therapeutic approaches in HCC treatment, including molecular‐targeted therapy, targeted radionuclide therapy, epigenetic modification‐based therapy, immunotherapy and gene therapy, have been discussed. Many researchers have tried to push forward current treatment protocols through combination of different approaches. However, despite promising technological advances, novel modalities have only managed to increase the overall survival rate and progression‐free survival rate by a few months. This reflects the fact that only the tip of the iceberg has been exposed, and we need to extend our knowledge in order to widen our horizons with respect to treatment modalities, perhaps before starting a new clinical trial.

It is clear that improving our understanding of the molecular mechanisms of (i) liver carcinogenesis, (ii) drug resistance (iii) and tumour metastasis should be the priority of future studies. With the advent of bioinformatics and computational tools, the molecular mechanisms underlying these areas of concern can be pinpointed with greater ease and accuracy than ever before. Bioinformatics, such as mapping genetic polymorphisms, analysing the interactions between gene products and predicting biochemical alterations, could provide precise and comprehensive insights into finding new target genes. In addition, computational methods could facilitate the design of advanced recombinant antibodies with higher affinities and reduced off‐target binding as novel bio‐therapeutics.

A crucial bottleneck to progress could occur in the polymorphic differences between species and differences in metabolic pathways and immune systems between human and animal models that prevent accurate interpretation of preclinical models. This could result in the failure of many drugs during their early development and clinical trials despite promising results in the preclinical phase. However, in recent years, various liver‐derived in vitro models such as organoids, microfluidic liver biochips, bio‐printed micro‐patterned co‐cultures and multi‐organ‐on‐a‐chip systems have been developed.^107^ These technologies are going towards bridging the gap between preclinical and clinical studies. As shown in Figure 3, a growing trend in the number of studies over the past two decades and successive FDA approvals for the new medications demonstrates that these efforts are bearing fruit and that researchers are on the right track. The upper box illustrates the starting point of important clinical trials in HCC treatment, which in addition to upward trend, displays the focus of the studies on targeted approaches. The middle box indicates the FDA approval dates for novel medications prescribed for HCC treatment. Box 2 shows that the number of approved medications was remarkably increased during the last five years. Finally, the bottom box specifies the date and the name of the first FDA‐approved landmark medications in different cancer therapeutic approaches, and perhaps the most promising event in this context is the recent FDA approval for two combination therapies for the first time (nivolumab in combination with ipilimumab in 11 March 2020; and atezolizumab a PD‐L1 checkpoint inhibitor, employed with bevacizumab, a VEGF‐A monoclonal antibody, in May 29, 2020). Given the positive feedback from such combination therapies, this approach seems to be a promising prospect for HCC treatment in the future.

## CONFLICT OF INTEREST

No conflict of interest can be disclosed. The authors declare that they have no competing interests.

## AUTHOR CONTRIBUTION

**Bahare Shokoohian:** Conceptualization (lead); Writing‐original draft (supporting). **Babak Negahdari:** Conceptualization (equal); Writing‐review & editing (equal). **Hamidreza Aboulkheyr Es:** Conceptualization (equal); Writing‐review & editing (equal). **Manuchehr Abedi‐Valugerdi:** Writing‐review & editing (supporting). **Kavei Baghaei:** Writing‐review & editing (equal). **Tarun Agarwal:** Writing‐review & editing (equal). **Tapas Kumar Maiti:** Writing‐review & editing (equal). **Moustapha Hassan:** Writing‐review & editing (equal). **Mustapha Najimi:** Writing‐review & editing (equal). **Massoud Vosough:** Conceptualization (lead); Writing‐review & editing (equal).

## Supporting information

Table S1‐S5

## Data Availability

Data openly available in a public repository that issues datasets with DOIs
