# Supplementary material for: Advanced therapeutic modalities in hepatocellular carcinoma: Novel insights
Source: J Cell Mol Med. 2021 Aug 23;25(18):8602–14. doi: 10.1111/jcmm.16875 (PMC8435417; doi:10.1111/jcmm.16875)
Supplement: Supplementary file 1 — Table S1‐S5 [file JCMM-25-8602-s001.docx]

***Supplementary Material***

*Review*

**Advanced Therapeutic Strategies in Hepatocellular Carcinoma: Novel Insights**

Bahare Shokoohian^1,2^, Babak Negahdari^1,^*****, Hamidreza Aboulkheyr Es^3^, Manuchehr Abedi-Valugerdi^4^, Kaveh Baghaei^5^, Tarun Agarwal^6^, Tapas Kumar Maiti^6^, Moustapha Hassan^4^, Mustapha Najimi^7,^*****, Massoud Vosough^2, 4,^*****

1. **Molecular Targeted Therapy in HCC**

Supplementary Table 1 | **FDA approved multikinase inhibitors for the treatment of HCC**

| **Agent** | **Targets** | **Interval level** | **Introducing clinical trial** | **Introducing date** | **FDA approval date** |
| --- | --- | --- | --- | --- | --- |
| Sorafenib | C-Raf B-Raf, VEGFR-1, -2, -3 PDGFR-β, c-Kit, CD135, RET1,2 | First Line | SHARP & Asia-Pacific | 2005 | 2007 |
| Lenvatinib | VEGFR-1. -2, -3, PDGFR-α, FGFR-1, -2, -3, -4, c-Kit, RET | First Line | REFLECT | 2013 | 2018 |
| Regorafenib | VEGFR-1, -2, -3, TIE2, PDGFR-β, FGFR, KIT, RET,  C-Raf, B-Raf | Second Line | RESORCE | 2013 | 2016 |
| Cabozantinib | c‐Met, VEGFR-2, AXL, RET, TIE2, CD135 | Second Line | CELESTIAL | 2013 | 2019 |
| Ramucirumab | VEGFR-2 | Second Line | REACH-2 | 2015 | 2019 |

VEGFR: vascular endothelial growth factor receptor; PDGFR: platelet-derived growth factor receptor; FGFR: fibroblast growth factor receptor;

1. **Targeted Radionuclide Therapy in HCC**

Supplementary Table 2 | **Clinical trials of targeted radionuclide therapy for the treatment of HCC**

| **Radionuclide agent** | | **Phase** | **Outcome** | **Ref** |
| --- | --- | --- | --- | --- |
| **Radioembolization** | ^90^Y-conjugated glass or resin microparticles | Phase III | A higher tumor radiation-absorbed dose and a better OS and disease control in individuals with unresectable advanced HCC. | 1  (NCT01482442) |
|  | ^90^Y-glass microspheres (TheraSphere) | Phase II | An effective treatment in intermediate to advanced HCC, particularly in the case of portal vein thrombosis with 9.6% complete responses. | 2  (NCT00910572) |
|  | ^131^I-lipiodol | Phase III | Significantly decrease in the rate of intrahepatic tumor recurrence, but failed to improve OS. | 3  (NCT00870558) |
|  | ^188^Re-HDD lipiodol | Phase II | Tumor size reduction in 25% of patients and a progression-free disease in 53% of patients compared to control group. | 4 |
|  | ^188^Re-SSS lipiodol  ^131^I-lipiodol | Phase I | Further increase in survival rate in ^131^I-lipiodol treated rats compared to the mixture of Re-SSS lipiodol and I-lipiodol or Re-SSS alone. | 5  (NCT01126463) |
| **Radioimmunotherapy** | ^131^I-labelled metuximab  (Licartin) | Phase IV | Better localization of licartin in liver tumors than other tissues, significantly increase in 1-year survival rate (79.47% *vs.* 65.59%) and TTP (6.82 *vs.* 4.7 months) compared to the control group. | 6 |
|  | ^131^I-labelled metuximab  (Licartin) | Phase II | A significantly higher five-year recurrence-free survival (43.4% *vs.* 21.7%) and five-year OS 61.3% in the ¹³¹I-metuximab group *vs* 35.9% in the control group. | 7  (NCT00819650) |

OS: overall survival; TTP: time-to-progression;

1. **Epigenetic alteration-based therapies in HCC**

Supplementary Table 3 | **Clinical trials of epigenetic modification-based therapies for the treatment of HCC**

| Target | Drug | Phase | Description / Outcome | Clinical Trial Identifier |
| --- | --- | --- | --- | --- |
| DNMT | Decitabine | Phase I/II | Hepatic arterial administration of decitabine could increase the efficacy of subsequent treatment with immune checkpoint inhibitors | NCT02316028 |
| DNMT | Guadecitabine | Phase II | Guadecitabine inhibited tumor growth and induced re-expression of silenced TSGs, alone or in combination with sorafenib | NCT01752933 |
| DNMT | Guadecitabine | Phase I | A study designed to determine side effects and best dose of guadecitabine, alone or in combination with durvalumab | NCT03257761 |
| DNMT | EGCG | Phase I | A study designed to determine side effects and best dose of defined green tea catechin extract in preventing liver cancer in cirrhosis patients. The gallic acid moiety of EGCG blocks the DNMT1 | NCT03278925 |
| HDAC  EGFR  HER2 | CUDC-101 | Phase I | CUDC-101 efficiently blocked tumor growth *in vitro* and *in vivo* and had an acceptable safety profile in patients | NCT01171924 |
| HDAC | Vorinostat | Phase I | The addition of vorinostat to sorafenib led to toxicities in most patients, requiring dose modifications | NCT01075113 |
| HDAC | Panobinostat | Phase I | A study designed to determine side effects and best dose of panobinostat in combination with sorafenib | NCT00873002 |
| HDAC | Panobinostat | Phase I | A study designed to determine side effects and best dose of panobinostat in combination with sorafenib | NCT00823290 |
| HDAC | Tefinostat | Phase I/II | A study designed to determine side effects and best dose of tefinostat | NCT02759601 |
| HDAC | Belinostat | Phase I/II | 45% of patients achieved tumor stabilization, and HR23B was identified as a response biomarker | NCT00321594 |
| HDAC | Resminostat | Phase II | Induced more epithelial phenotype and increased sorafenib-induced cell death; in combination with sorafenib prolonged PFS and OS in HCC patients | NCT00943449 |
| C/EBP-a | MTL-CEBPA | Phase I | 50% of patients achieved stable disease; it showed an acceptable safety profile and potential synergistic efficacy with TKIs in HCC | NCT02716012 |
| MYC PDGFR-α CDK4/6  BCL2 | miR-RX34 | Phase I | MRX34 treatment with dexamethasone premedication confirmed a manageable toxicity profile but serious immune-mediated AEs resulted in death of 4 patients and early closure of the study | NCT01829971 |

DNMT: DNA methyl transferase; HDAC: Histone deacetylase; TSG: tumor suppressor gene; EGCG: Epigallocatechin gallate; HER2: human epidermal growth factor receptor 2; C/EBP: CCAAT-enhancer-binding protein;

1. **Gene therapy approaches in HCC**

Supplementary Table 4 | **Clinical trials using viral vectors for the HCC treatment.**

| Viral vector Used | Clinical Trial Phase | Patients Enrolled status | Clinical Trial Identifier |
| --- | --- | --- | --- |
| rAd-p53 | Phase II | Advanced HCC patients with diabetes | NCT02561546 |
| rAd-p53 | Phase II | Advanced HCC patients with unresectable cancer | NCT02509169 |
| rAd-p53 | Phase II | Advanced HCC patients with unresectable cancer | NCT02418988 |
| Ad5-CMV-p53 | Phase I | Advanced HCC patients with unresectable cancer | NCT00003147 |
| rAd-p53 | Phase I/II | Patients with unresectable liver metastases of colorectal carcinoma and other solid tumors | NCT02842125 |
| rhAdV5-p53 & GM-CSF & B7-1 | Phase III | Advanced HCC patients with unresectable cancer | NCT01869088 |
| ADV-TK | Phase II | Advanced HCC patients with tumor diameter >5 cm  who can undergo LT | NCT02202564 |
| ADV-TK | Phase II | Intermediate or advanced HCC patients who can undergo LT | NCT00300521 |
| ADV-TK | Phase III | Advanced HCC Patients who can undergo LT | NCT03313596 |
| ADV-HSV-TK (TK99UN) | Phase I | Advanced HCC patients that were not responsive to curative therapy | NCT00844623 |
| AdV-AFP | Phase I/II | Patients with stage II, stage IIIA, stage IIIB, or stage IVA liver cancer | NCT00093548 |
| hAdV-AFP | Phase I/II | Patients with locoregionally pre-treated HCC | NCT00669136 |
| Ad5-CEA (ETBX-011)  Ad5-Brachyury (ETBX-051)  Ad5-MUC1 (ETBX-061) | phase Ib/II | Advanced HCC patients with unresectable and untransplantable cancer | NCT03563170 |
| rhAdV type-5 (H101) | Phase III | HCC patients with unresectable tumors at BCLC A-B stage | NCT03780049 |
| rhAdV type-5 (H101) | Not Applicable | HCC patients with single lesion ≤ 3 cm in diameter | NCT03790059 |
| JX-594 (Pexa-Vec) | Phase II | Advanced HCC patients with unresectable cancer | NCT01171651 |
| JX-594 (Pexa-Vec) | Phase III | Advanced HCC patients who have not received prior therapy | NCT02562755 |
| JX-594 (Pexa-Vec) | Phase II | HCC patients with unresectable cancer | NCT00554372 |
| JX-594 (Pexa-Vec) | Phase I | HCC patients resistant to standard treatment | NCT00629759 |
| JX-594 (Pexa-Vec) | Phase IIb | Advanced HCC patients who have failed sorafenib | NCT01387555 |
| JX-594 (Pexa-Vec) | Phase II | Advanced HCC patients who have not been treated with sorafenib | NCT01636284 |
| p53MVA vaccine | Phase I | Advanced HCC patients with unresectable cancer | NCT02432963 |
| Lenti- ET140202 | Phase I/II | AFP positive/ HLA-A2 positive advanced HCC patients | NCT03998033 |
| Lenti- ET140202 | Early Phase I | AFP positive/ HLA-A2 positive advanced HCC patients with poor overall prognosis | NCT03965546 |
| Lenti- ET1402L1 | Early Phase I | patients with AFP positive HCC | NCT03888859 |
| Lenti- ET1402L1 | Phase I | patients with AFP positive HCC | NCT03349255 |
| Lenti-AFP (TCR gene) | Phase I | Advanced HCC patients with unresectable cancer | NCT03971747 |
| Lenti-c-Met/PD-L1(TCR gene) | Early Phase I | Patients with primary HCC | NCT03672305 |

rAd-p53: recombinant adenovirus expressing p53; ADV-TK: thymidine kinase-deficient virus; ADV-HSV-TK: adenovirus-containing herpes simplex virus thymidine kinase; Pexa-Vec: pexastimogene devacirepvec; LT: Liver Transplantation; p53MVA vaccine: modified vaccinia virus Ankara vaccine expressing p53, Lenti- ET140202: Lentivirus encoding an anti-AFP (ET140202) expression construct, TCR: T cell receptor.

Supplementary Table 5 | **List of studies using suicide genes for HCC treatment.**

| Enzyme/ Prodrug pairs | Vector/Delivery system | Enzyme promoter | Cell platform | Brief explanation | Ref |
| --- | --- | --- | --- | --- | --- |
| HSVtk/GCV | Adenovirus/Viral transduction | AFP | *In vivo*  (clinical trial phase I) | Intratumoral injection of Ads expressing HSVtk with systemic GCV to HCC patients was safe but had no partial responses. | ^8^ |
| HSVtk/GCV | Adenovirus/Viral transduction | - | *In vivo*  (clinical trial phase II) | The PFS and OS rates in LT + ADV-TK/GCV patients at 3 years were significantly higher than those in the LT-only group. | ^9^ |
| HSVtk/GCV | Liposomal transfection | Survivin | HepG2 & LO2 | HSVtk showed cancer-specific expression under the control of the survivin promoter. | ^10^ |
| HSVtk/GCV | Bacterial plasmid/ Ultrasound lipid microbubble-mediated transfection | - | HepG2 | Pre-treatment with ATRA elevated the expression of connexin32 and enhanced the bystander effect of HSVtk/GCV. | ^11^ |
| HSVtk/GCV | Adenovirus/Viral transduction | PEPCK + ApoE enhancer | Hep3B & HDF | By adding the PEPCK promoter and ApoE enhancer, HSVtk expression was dedicated to liver cells. Transduction rate also increased through changes in the rhAdV type-5 backbone. | ^12^ |
| HSVtk/GCV | Shaped controlled magnetic mesoporous silica nanoparticles (M-MSNs) | - | HepG2 | TK and GCV were loaded into M-MSNs, and then were injected and MRI-guided to the tumor site in mouse models. | ^13^ |
| CD/5-FC | Retrovirus/Viral transduction | - | HuH-7, HLF, HAK1-B, KYN-2, KIM-1 &  TR-BME-2 | TR-BME-2 cells were transfected with CD, and the inhibitory effect of produced 5-FU was evaluated in HCC cell lines. | ^14^ |
| CD/5-FC | Protein/Cell-penetrating peptide (R9) | - | HepG2 & U251 | To avoid risks of gene transfer a cell-penetrating peptide (R9) was fused to bacterial CD, and to improve the turnover rate of 5-FC, the CD was subjected to site mutagenesis. | ^15^ |
| CD/5-FC TK/GCV | Cationic microbubbles conjugated with αVβ3 integrin antibody | VEGF | HepG2 & LO2 | A double suicide gene system under control of the VEGF promoter selectively kills HCC cells, *in vitro* and *in vivo.* | ^16^ |
| CD/5-FC TK/GCV | Adenovirus/Viral transduction | VEGF | BEL-7402, HepG2 & HUVEC | A double suicide gene system under control of the VEGF promoter selectively kills HCC cells, *in vitro* and *in vivo.* | ^17^ |
| PNP/FP | pcDNA3.1(+)/Ultrasound lipid nanobubble-mediated transfection | - | HepG2 & SMMC7721 | Nanobubbles containing pcDNA3.1 (+)/PNP transfected HCC cells and bystander effect was also exhibited. | ^18^ |
| PNP/FP  HSVtk/GCV | Adenovirus/Viral transduction | - | HepG2 & Hep3B | To compare the efficacy and bystander effect of PNP/FP with HSVtk/GCV, HCC cells were transduced with both systems and indicated the superiority of PNP/FP bystander effect induction. | ^19^ |
| PNP/FP  CD/5-FC | Retrovirus/Viral transduction | AFP +  EII enhancer (EIIAFP) | HepG2, Huh-7, THLE-2, MCF-7, T-47D, U-87, HEK293 | CD/5-FC and PNP/FP systems under the control of AFP promoter and EII enhancer selectively kill HCC cells, *in vitro* and *in vivo.* | ^20^ |

HSVtk/GCV: Herpes simplex virus thymidine kinase/ ganciclovir; PEPCK: Phosphoenolpyruvate carboxykinase; CD/5-FC: Cytosine deaminase/ 5-fluorocytosine; R9: Polyarginine; PNP/FP: Purine nucleoside phosphorylase/fludarabine phosphate; EII enhancer: Hepatitis B virus II enhancer.

1. **References**

1. Hermann A-L, Dieudonné A, Ronot M, et al. Relationship of tumor radiation–absorbed dose to survival and response in hepatocellular carcinoma treated with transarterial radioembolization with 90Y in the SARAH study. *Radiology*. 2020:191606.

2. Mazzaferro V, Sposito C, Bhoori S, et al. Yttrium‐90 radioembolization for intermediate‐advanced hepatocellular carcinoma: a phase 2 study. *Hepatology*. 2013;57(5):1826-1837.

3. Dumortier J, Decullier E, Hilleret M-N, et al. Adjuvant intraarterial lipiodol or 131I-lipiodol after curative treatment of hepatocellular carcinoma: a prospective randomized trial. *Journal of Nuclear Medicine*. 2014;55(6):877-883.

4. Bernal P, Raoul J-L, Stare J, et al. International Atomic Energy Agency-sponsored multination study of intra-arterial rhenium-188-labeled lipiodol in the treatment of inoperable hepatocellular carcinoma: results with special emphasis on prognostic value of dosimetric study. Seminars in Nuclear Medicine: Elsevier; 2008. p. S40-S45.

5. Garin E, Rakotonirina H, Lejeune F, et al. Effect of a 188Re-SSS lipiodol/131I-lipiodol mixture, 188Re-SSS lipiodol alone or 131I-lipiodol alone on the survival of rats with hepatocellular carcinoma. *Nuclear medicine communications*. 2006;27(4):363-369.

6. Ma J, Wang J-H. 131 I-Labeled-Metuximab plus transarterial chemoembolization in combination therapy for unresectable hepatocellular carcinoma: results from a multicenter phase IV clinical study. *Asian Pacific Journal of Cancer Prevention*. 2015;16(17):7441-7447.

7. Li J, Xing J, Yang Y, et al. Adjuvant 131I-metuximab for hepatocellular carcinoma after liver resection: a randomised, controlled, multicentre, open-label, phase 2 trial. *The Lancet Gastroenterology & Hepatology*. 2020.

8. Sangro B, Mazzolini G, Ruiz M, et al. A phase I clinical trial of thymidine kinase-based gene therapy in advanced hepatocellular carcinoma. *Cancer gene therapy*. 2010;17(12):837-843.

9. Zhu R, Weng D, Lu S, et al. Double-dose adenovirus-mediated adjuvant gene therapy improves liver transplantation outcomes in patients with advanced hepatocellular carcinoma. *Human gene therapy*. 2018;29(2):251-258.

10. Qu L, Wang Y, Gong L, Zhu J, Gong R, Si J. Suicide gene therapy for hepatocellular carcinoma cells by survivin promoter-driven expression of the herpes simplex virus thymidine kinase gene. *Oncology reports*. 2013;29(4):1435-1440.

11. Wu L, Zhou WB, Shen F, et al. Connexin32‑mediated antitumor effects of suicide gene therapy against hepatocellular carcinoma: In vitro and in vivo anticancer activity. *Molecular medicine reports*. 2016;13(4):3213-3219.

12. Kim Y-H, Kim KT, Lee S-J, et al. Image-aided suicide gene therapy utilizing multifunctional hTERT-targeting adenovirus for clinical translation in hepatocellular carcinoma. *Theranostics*. 2016;6(3):357.

13. Wang Z, Chang Z, Lu M, et al. Shape-controlled magnetic mesoporous silica nanoparticles for magnetically-mediated suicide gene therapy of hepatocellular carcinoma. *Biomaterials*. 2018;154:147-157.

14. Torimura T, Ueno T, Taniguchi E, et al. Interaction of endothelial progenitor cells expressing cytosine deaminase in tumor tissues and 5‐fluorocytosine administration suppresses growth of 5‐fluorouracil‐sensitive liver cancer in mice. *Cancer science*. 2012;103(3):542-548.

15. Wang W, Zhang N, Zhao T, Liu M, Zhang T, Li D. Inhibition of tumor growth by polyarginine-fused mutant cytosine deaminase. *Applied biochemistry and biotechnology*. 2015;175(3):1633-1643.

16. Li J, Zhou P, Li L, et al. Effects of cationic microbubble carrying CD/TK double suicide gene and αvβ3 integrin antibody in human hepatocellular carcinoma HepG2 cells. *PloS one*. 2016;11(7).

17. Wu K, Yang L, Huang Z, Zhao H, Wang J, Xu S. A double suicide gene system driven by vascular endothelial growth factor promoter selectively kills human hepatocellular carcinoma cells. *Oncology letters*. 2016;11(5):3152-3160.

18. Zhang B, Chen M, Zhang Y, Chen W, Zhang L, Chen L. An ultrasonic nanobubble-mediated PNP/fludarabine suicide gene system: A new approach for the treatment of hepatocellular carcinoma. *PloS one*. 2018;13(5).

19. Krohne TU, Shankara S, Geissler M, et al. Mechanisms of cell death induced by suicide genes encoding purine nucleoside phosphorylase and thymidine kinase in human hepatocellular carcinoma cells in vitro. *Hepatology*. 2001;34(3):511-518.

20. Lai Y, Lin C, Chen S, Tai C. Tumor-specific suicide gene therapy for hepatocellular carcinoma by transcriptionally targeted retroviral replicating vectors. *Gene therapy*. 2015;22(2):155-162.
